# Composition of *Anopheles* Species Collected from Selected Malarious Areas of Afghanistan and Iran

**Published:** 2017-09-08

**Authors:** Helen Hoosh-Deghati, Navid Dinparast-Djadid, Vahideh Moin-Vaziri, Hoda Atta, Abbas Ali Raz, Seyyed Javad Seyyed-Tabaei, Naseh Maleki-Ravasan, Hamzeh Alipour, Sedigheh Zakeri, Eznollah Azar-Gashb

**Affiliations:** 1 Department of Parasitology, School of Medicine, Shahid Beheshti University of Medical Sciences, Tehran, Iran; 2 Malaria and Vector Research Group, Biotechnology Research Center, Pasteur Institute of Iran, Tehran, Iran; 3 Malaria Control, Word Health Organization, Eastern Mediterranean Regional Office, Cairo, Egypt

**Keywords:** *Anopheles*, Morphological identification, Afghanistan, Iran

## Abstract

**Background:**

Malarious areas in Iran are close to Afghanistan and Pakistan that urge the researchers to extend their knowledge on malaria epidemiology to the neighboring countries as well. Vectorial capacity differs at species or even at population level, the first essential step is accurate identification of vectors. This study aimed to identify *Anopheles* species composition in selected malarious areas of Afghanistan and Iran, providing further applied data for other research in two countries.

**Methods:**

Adults *Anopheles* spp. were collected from four provinces in Afghanistan (Badakhshan, Herat, Kunduz, Nangarhar) by pyrethrum spray catch, hand collection methods through WHO/EMRO coordination and from Chabahar County in Iran by pyrethrum spray catch method. Identification was performed using reliable identification key.

**Results:**

Totally, 800 female *Anopheles* mosquitos, 400 from each country were identified at species level. *Anopheles* composition in Afghanistan was *An. superpictus*, *An. stephensi* and *An. hyrcanus*. Most prevalent species in Badakhshan and Kunduz were *An. superpictus*, whereas *An. stephensi* and *An. hyrcanus* were respectively found in Nangarhar and Heart. *Anopheles* species in Chabahar County of Iran were *An. stephensi*, *An. fluviatilis*, *An. culicifacies* and *An. sergentii*. The most prevalent species was *An. stephensi.*

**Conclusion:**

Current study provides a basis for future research such as detection of *Plasmodium* infection in collected samples which is on process by the authors, also for effective implementation of evidence-based malaria vector intervention strategies.

## Introduction

Malaria is known as one of the six important infectious diseases based on WHO (1). It is caused by the protozoon *Plasmodium* spp and transmitted by female *Anopheles* mosquitoes (2). *Anopheles* spp belongs to Culicidae family (Diptera) with 460 or so different species that 70 of them have the potential of being malarial vectors, however, around 36 species are considered as main ones (3, 4, 5). About 198 million cases of malaria occurred in 2013, leading to 584000 deaths (6). Globally, around 3.2 billion people are at risk of being infected with malaria and 1 in 1000 is at high risk of getting malaria in year (1). In Eastern Mediterranean regions (EMR), more than 10 million clinical malaria cases occur annually with 50 thousands death. Generally 287 million which is approximately 60% of Eastern Mediterranean population is at the risk of malaria infection (1, 7, 8).

Malaria is of great importance from public health concerns in Iran, more than 2 million people live at high risk regions (7). Iran with population of more than 78 million people is located in West Asia sharing a border of 1458 and 936km respectively with Iraq from the west, Afghanistan, and Pakistan from the east (9). Malaria is endemic in the South and Southeast of Iran, where more than 90 percent of malaria cases are reported (10, 12). These regions are bordered with Pakistan and Afghanistan which malaria cases due to *P. falciparum* and *P. vivax* are prevalent. Malaria has an unstable pattern varying between 42% up 60% of total malaria cases with two seasonal peaks mostly in spring and autumn after the rainy seasons in the provinces located in South-East of Iran comprising Sistan and Baluchistan, Hormozgan and the tropical part of Kerman (13). Recently a notable reduction has been observed in malaria cases due to the report of Ministry of Health and Medical Education of Iran, which has led WHO to categorize Iran in the elimination phase (14). Nationwide campaign malaria elimination was launched by Iranian Government in 2010 in epidemic regions with the goal of becoming malaria-free country by 2025 (12, 14).

Vector control is the most efficient way to decrease the malaria transmission at the community level. Regarding to the recent Iranian mosquitoes checklist, seven different genera, 64 species and 3 subspecies are reported (15). Seven species of the genus *Anopheles* including *An. culicifacies* Giles s.l., *An. dthali* Patton, *An. fluviatilis* James s.l., *An. maculipennis* Meigen s.l., *An. sacharovi* Favre, *An. stephensi* Liston, and *An. superpictus* Grassi are identified as malarial vectors in Iran. Six species are known as its vectors in southeast of Iran: *An. culicifacies*, *An. stephensi*, *An. dthali*, *An. fluviatilis*, *An. pulcherrimus*, and *An. superpictus*. Among them, *An. stephensi* is the main vector of malaria in the area and considerably prefers human blood (16, 13, 12).

In Afghanistan, approximately half of the population is at the risk of malaria. There are at least six malaria vectors in Afghanistan: *Anopheles superpictus*, *An. culicifacies*, *An. hycranus*, *An. pulcherrimus*, *An. fluviatilis* and *An. stephensi* (17).

Full understanding of malaria vectors can be considered as a key factor in malaria control programmes. The first step in any control program is the vector species identification. Moreover, malaria endemic areas in Iran are close to Afghanistan and Pakistan, urging us to extend our research to the neighboring countries as well.

*Anopheles* species were collected from selected malarious areas of Afghanistan (Badakhshan, Herat, Kunduz, and Nangarhar) located in high risk areas (18, 19) by WHO.

This study aimed to identify *Anopheles* species composition in selected malarious areas of Afghanistan and Iran, providing further applied data for other research in two countries. Results presented here reflect the composition of *Anopheles* species of mentioned collection as a part of ongoing project. It could provide also useful information in planning and implementing of an effective program for vector control during elimination phase in Iran and control phase in Afghanistan for the national malaria control program.

## Materials and Methods

### Study areas

**Fig. 1.**
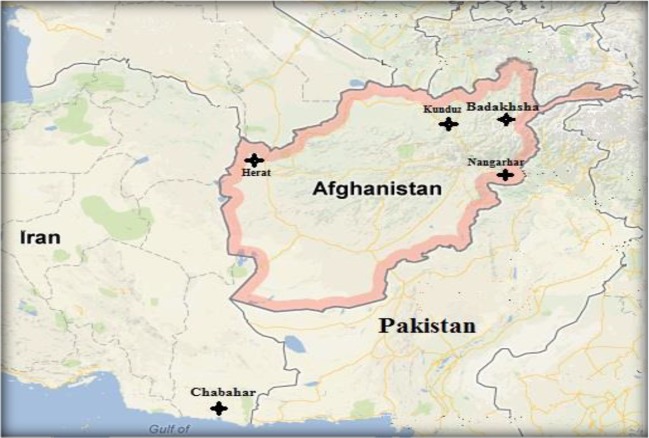
Map of Iran and Afghanistan, indicating the location of the study area in Chabahar County (marked with an asterisk) situated in the south of Sistan and Baluchistan in Iran and Herat, Kunduz, Badakhshan, Nangarhar provinces in Afghanistan.

Afghanistan: Afghanistan is a landlocked country in south central Asia (33° 00′ N, 65° 00′ E). Its climate is arid to semiarid with frequent sand storms. The northern and southeastern areas of the country have very rugged mountain terrains (Hindu Kush), while the western and southern parts are flat deserts and plains. Climate varies according to the elevation and location. In general, malaria occurs at altitude below 2,000m and is the most prevalent in snow-fed river valleys and rice growing areas (20). Samples were collected from four counties, located in different geographical sites of Afghanistan, North-East (Badakh shan and Kunduz), South-East (Nangarhar) and Western part close to Iran boundary (Herat) (Fig. 1).

Iran: Sistan and Baluchistan Province as the main malarious area is located in the Northern coast of Persian Gulf and Oman Sea, where the weather is warm and humid enough for *Anopheles* species to be activated throughout the year (7).

This study took place in Chabahar County (25° 17′ N, 60° 38′ E), bounded by Pakistan in the east, Hormozgan Province in the west, Kerman Province in the north, and Oman Sea in the south. It has a warm and humid weather in summer and temperate in winter. The average maximum and minimum temperature are respectively 34 °C and 10.5 °C in July and January. Latitude from the sea level is 7m. The annual rainfall is low mainly in spring and autumn so that it has a desert climate. However it has high humidity due to neighboring sea (Fig. 1) (21).

### Mosquito collection and identification

Adult *Anopheles* spp were collected on August 2011 through WHO/EMRO coordination from four provinces of Afghanistan (Badakhshan, Herat, Kunduz, Nangarhar) by pyrethrum spray catch, hand collection method using manual aspirator and from Chabahar County in Iran by pyrethrum spray catch method (Table 1). The sampling sites are indicated on Fig. 1. The species identification was performed by using reliable identification key (22) under Leica EZ4D (16X) stereomicroscope.

**Table 1.** Details of collected specimens based on study areas and collection methods: PSC: Pyrethrum Spray Catch, HC: Hand Collection by manual aspirator, HC: Hand Collection, HH: Human House, AH: Animal House

	**Province**	**Village**	**Collection methods and Sites**	**Number of Mosquitoes**	***Anopheles* spp**	**Total number of species**	**Total**
**Afghanistan**			PSC-HH (Indoor)	70	*An. hyrcanus*		400
		
	Herat	Haja Sourma	PSC-AH (Indoor)	30		110 (27.5%)	

		Pol-e taracheh	PSC-HH (Indoor)	55	*An. stephensi*		
			
	Nangarhar	Ali khan		55		110 (27.5%)	

	Badakhshan	Darewazer	PSC-AH (Indoor)	39	*An. superpictus*	100 (25%)	

			HC-HH (Indoor)	37			

			PSC-HH (Indoor)	24			

	Kunduz	Gerghiz	PSC-HH (Indoor)	80	*An. superpictus*	80 (20%)	

**Iran**	Chabahar	Owraki	PSC-HH (Indoor)	100	*An. stephensi* *An. fluviatilis*	379 (94.75%) 11 (2.75%)	400

		Balochadam	PSC-HH (Indoor)	300	*An. culicifacies* *An. sergentii*	6 (1.5%) 4 (1%)	

## Resuts

### Morphological identification of Afghanistan Anophelinae mosquitoes

**Fig. 2.**
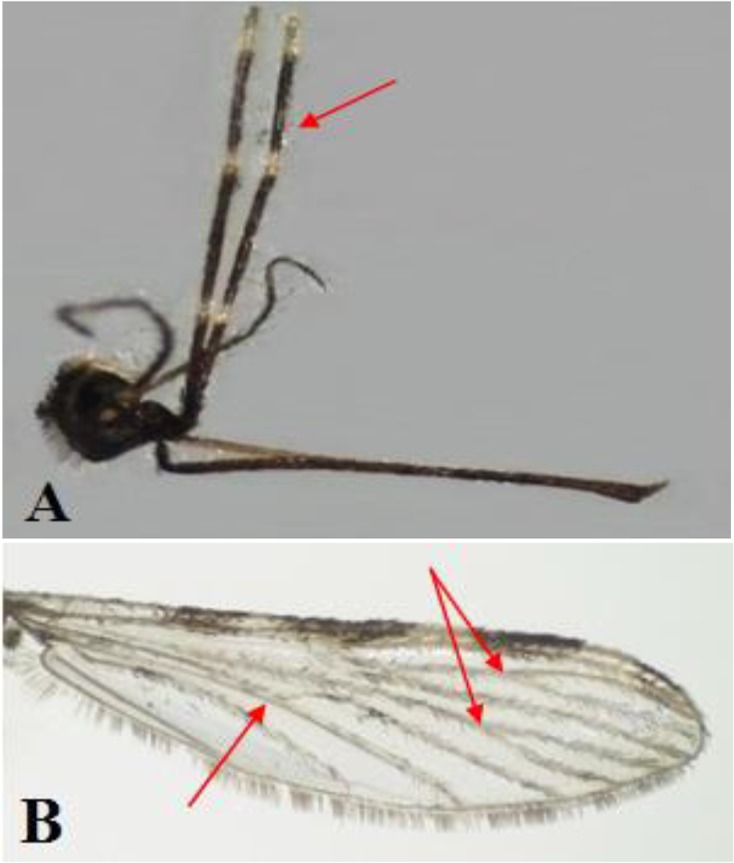
*Anpheles superpictus*, A: Head with long palps B: No dark spot at point of bifurcation of 5th vein and division part of the second and forth vein to the end of wing is equal

**Fig. 3.**
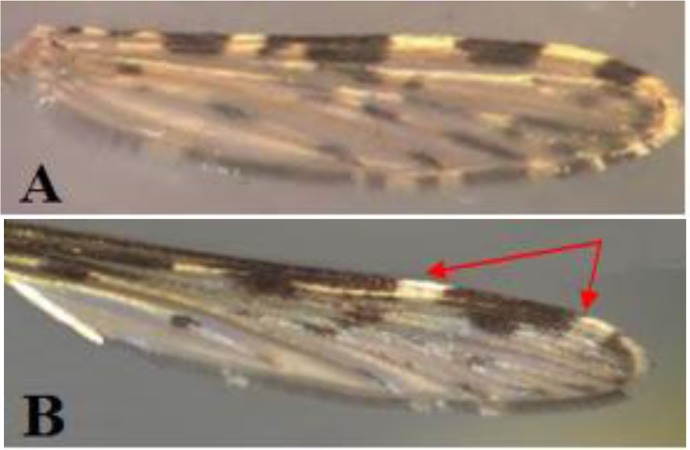
A: existence at least dark spot at the upper margins of the swings, which is common in all *Anopheles* except *An. hyrcanus* B: *An. hyrcanus*, fore margin of the wing with two small white spots

A total of 400 adults female *Anopheles* were collected from Herat, Nangarhar, Badakhshan and Kunduz provinces of Afghanistan (Table 1). Among the collected specimens, 3 species were identified based on the morphological characteristics, 45% of total specimens belong to *An. superpictus* collected from Badakhshan and Kunduz. Totally 180 *An. superpictus* were collected from mentioned areas, with pyrethrum spray catch (PSC) and hand collection, among them 141 (78.3%) collected from human house. The major morphological characteristics of this species are having unusually long palps (Fig. 2A), no dark spots at point of bifurcation of 5th longitudinal vein and equal division part of the second and forth vein to the end of wing (Fig. 2B). Next species was *An. hyrcanus* which was gathered by PSC method from animal and human house in Herat. Totally 110 (27.5%) was identified, which mainly collected from human house (63%). In *An. hyrcanus* fore margin of wing has less than four light and four dark bands alternating (Fig. 3A), fore margin of the wing with two small white spots, one near the tip, the other about two thirds of the way from the root of the wing (Fig. 3B).

**Fig. 4.**
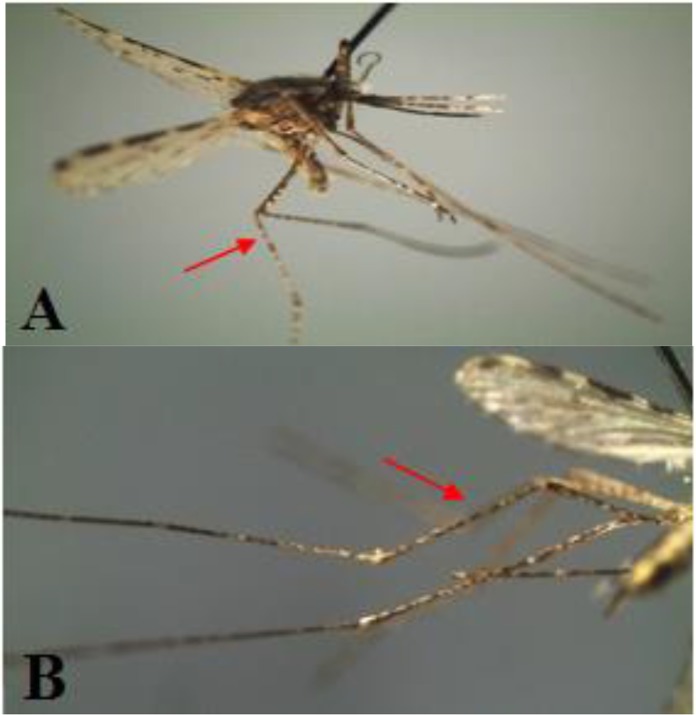
*Anopheles stephensi*, A, B: Femora and tibiae speckled with white spots

Third identified species was *An. stephensi* (27.5%), which was sampled from the human house of Nangarhar by PSC method. This species could be easily characterized by white spots which speckled on femora and tibiae (Fig. 4 A, B).

### Morphological identification of Anopheline mosquitoes of Chabahar County (Iran)

**Fig. 5.**
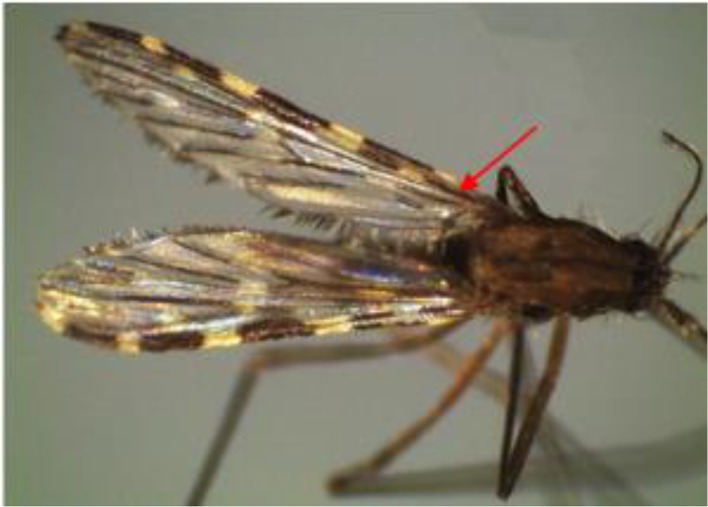
*Anopheles culicifacies*, first vein with a dark spot opposite the light spot at the base of costa

**Fig. 6.**
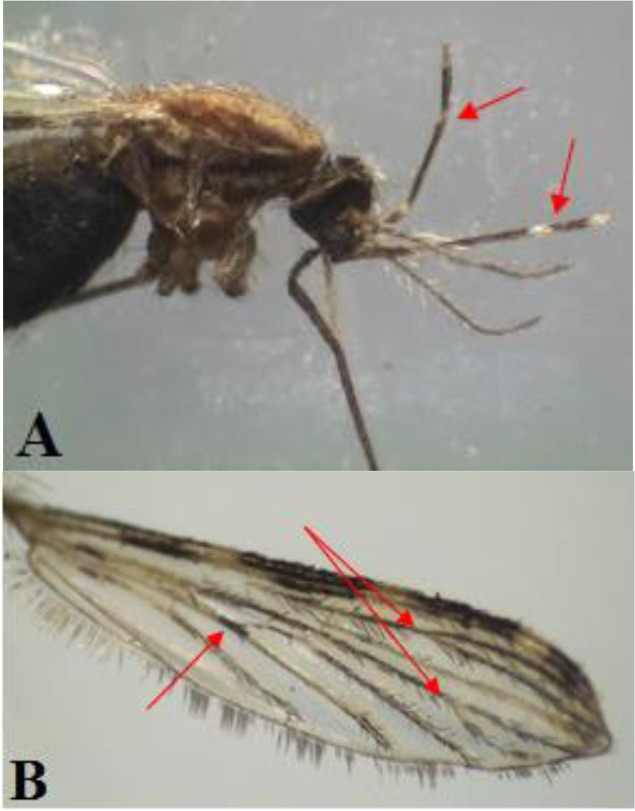
*Anopheles fluviatilis*, A: usual length of palps B: spot at the point of bifurcation of the 5th vein and division part of the second vein is farther than the forth vein to the end of wing

**Fig. 7.**
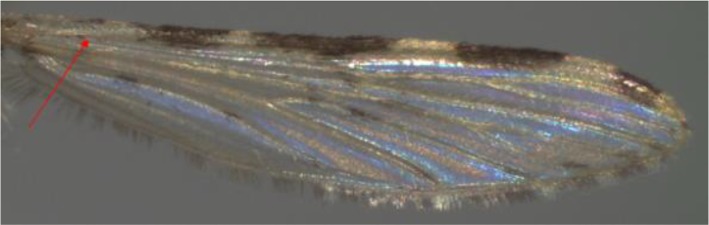
*Anopheles sergentii*, no dark spot on sub-costa

Morphological characteristic of 400 adults female *Anopheles* specimens collected from two villages of Chabahar County, Iran, revealed the presence of 4 different species (Table 1). Regardless the kind of species, all specimens was collected from human house and by PSC method. The most prevalent species among them was *An. stephensi* (94.75%), which its main morphology character was mentioned previously. The other species were *An. fluviatilis* (2.75%), *An. culicifacies* (1.5%) and *An. sergentii* (1%). *Anopheles culicifacies* has been identified based on the following main characters, existence of two fringe spots, no tuft of scales on anterior margin of mesonotum and finally existence of a dark spots opposite to the light spot at the costa base (Fig. 5). *Anopheles fluviatilis* has palps with usual length (Fig. 6A), dark spot at the point of bifurcation of the 5th vein and farther division part of the second vein than the forth vein with respect to the end of wing (Fig. 6B). *Anopheles sergentii* characteristics include more than two light spots on the posterior margin of fringe with pale base of subcostal and no dark spot of sub-costal opposite to the pale spot of costa (Fig. 7).

## Discussion

Malaria is widespread in the tropical and subtropical regions of the world and is also a public health problem in western extensions of the oriental zoogeographical region including Iran, Afghanistan and Pakistan. According to WHO report, 9 of the 97 countries with ongoing malaria transmission, including Iran, are classified as being in the malaria elimination phase. At this stage, interventions focus on the detecting all malaria cases, managing malaria foci, managing imported malaria cases and preventing onward transmission (23). Regarding the latest reports, it should be intentioned that, different *Anopheles* species even different population of one species, have different ecologies, biological attributes and vectorial capacities (24). Then, exact species identification is necessary for better understanding of their potential roles in malaria transmission (25, 12).

In Iran the most problematic malarious areas is south and southeast of the country, which highly affected by the epidemiology of malaria in neighboring countries, Afghanistan and Pakistan. The following reasons support this consumption 1. Afghanistan is in control phase of the malaria control, compared to Iran which is in elimination phase (14), 2. similarity of *Anopheles* fauna and human causative agents of malaria, both *P. falciparum* and *P. vivax* are prevalent in both countries and 3. importance of immigration from Afghanistan and Pakistan to malarious areas of Iran is the major routes of malaria transmission. This situation urges us to conduct current study, concentrating on morphological characters of *Anopheles* and their composition within selected areas of Afghanistan and Iran. Taxonomy that utilizes morphological characterization has been known as the gold standard method in identifing the mosquito species, which also have been used in current study for identification of five anopheline species in the study areas of Iran and Afghanistan, including known malaria vector species.

All the species which were identified during current study previously was reported in the checklist of Iranian mosquito fauna (15). The most prevalent species among our collected specimens from Chabahar was *An. stephensi* playing a dominant role in transmission of malaria in Persian Gulf as well in south of Iran (26, 27). This species is considered as an endophage and endophile mosquito. Sporozoite rate of this species was reported between 0.2 and 1.8% in south of Iran (26, 28) Other collected species were *An. culicifacies*, *An. fluviatilis* and *An. sergenti*; the first two types are also important as secondary vectors in the South and South-East of the country (3, 12).

Among secondary vectors, *An. culicifacies* can be regarded as potential vector, as it is greatly responsible for the epidemy of malaria in Sistan and Baluchistan Province (29). Sporozoite rate of this species is varied between 1–4.7% in south malarious areas of Iran (3, 12). Although it is mainly known as zoophilic *Anopheles*, its anthropophilic index is reported as 16.4% (30). *Anopheles culicifacies* has also a wide distribution in Asia and Indian subcontinent (31). *Anopheles fluviatilis*, distribution at altitude 50–1100m, along the foothills of the Zagros Mountains and extension to South and South-East of Iran as a secondary vector, its sporozoite rate was reported between 1.4–11% (3, 12). *Anopheles sergentii* is also prevalent in different provinces of Iran, acting as secondary vector and responsible for maintaining the parasite reservoir in malarious areas.

In Afghanistan, totally three species were identified among collected specimens, reported previously in mosquito fauna of this country (32). Comprising *An. superpictus*, *An. stephensi* and *An. hyrcanus*, which are all known vectors of malaria (33). *Anopheles superpictus* has a broad geographical distribution in Asia, Europe and Africa considered as a malaria vector in these areas (13, 12). It was collected from Badakhshan and Kunduz located in northeast of Afghanistan as it was reported previously (34). Moreover, the only species which was recently reported by Ahmad et al. from the Badakhshan was *An. superpictus* (35). Although during current study, this species was not found among the collected specimens of Chabahar, but it is commonly widespread in Iran in altitudes 50–2000m. Sporozoite rate of mentioned species is 0.65–4.7% in Iran (36, 3). *Anopheles stephensi* was collected mainly from Nangarhar Province in human house and mainly were blood-fed. As mentioned, this species is an important vector of human malaria in the Middle East and South Asia including Indo-Pakistan sub-continent (16). The third species in the study area was *An. hyrcanus*. It is a member of the Hyrcanus group comprising of 30 species, some of them are vectors of malaria, filariasis and arboviruses (37, 38). It is considered as the fauna of bloodsucking mosquitoes of Afghanistan (32, 17), also its potential role as malaria vector was reported from France, Turkey, Greece and Afghanistan (32, 39). This species has already been reported in the North and North-East of Iran (40), and is suspected to transmit malaria in Fooman District located in the north of Iran (41).

This study presents some limitations that may reflect the article. Mosquito collection was performed in a limited time and areas of Iran and Afghanistan due to the shortage of research time and lack of facilities, so although results are worthy enough for the other basic research, but could not regarded as a representative of a faunistic or eco-epidemiological study.

## Conclusions

Obtained results provide baseline information for other researchers and it reveals valuable information for designing further studies on vectorial capacity and detection of *Plasmodium* species and the status of insecticide resistance within collected *Anopheles* spp which is undergoing by the authors. Furthermore, it could be useful for designing, implementation and evaluation of local and regional evidence-based malaria control program.

## References

[1] World Health Organization (2014b) World Malaria Report 2014. WHO Press, Switzerland.

[2] Huldén L Huldén L (2014) Checklist of the family Culicidae (Diptera) in Finland. ZooKeys. (441): 47– 51. 10.3897/zookeys.441.7743 PMC4200447 25337007

[3] Hanafi-Bojd AA Azari-Hamidian SH Vatandoost H Charrahy Z (2011) Spatio-temporal distribution of malaria vectors (Diptera: Culicidae) across different climatic zones of Iran. Asian Pac J Trop Med. 4( 6): 498– 504. 21771707 10.1016/S1995-7645(11)60134-X

[4] Azizi MH Bahadori M (2013) Brief historical perspectives of malaria in Iran. Arch Iran Med. 16( 2): 131– 135. 23360639

[5] Stevenson J Norris D (2016) Implicating cryptic and novel Anophelines as malaria vectors in Africa. Insects. 8( 1): E1. 28025486 10.3390/insects8010001 PMC5371929

[6] Omima M Amany M (2015) Limited genetic diversity among *Plasmodium falciparium* isolates using nested PCR in Jazan Area, Saudi Arabia. Asian Pac J Trop Biomed. 5( 5): 407– 411.

[7] Chavshin AR Oshaghi MA Vatandoost H Hanafi-Bojd AA Raeisi A Nikpoor F (2014) Molecular characterization, biological forms and sporozoite rate of *Anopheles stephensi* in southern Iran. Asian Pac J Trop Biomed. 4( 1): 47– 51. 24144130 10.1016/S2221-1691(14)60207-0 PMC3819495

[8] Salmanzadeh Sh Foroutan-Rad M Khademvatan Sh Moogahi S Bigdeli Sh (2015) Significant decline of malaria incidence in Southwest of Iran (2001–2014). J Trop Med. 2015: 523767. 26649056 10.1155/2015/523767 PMC4663331

[9] World Health Organization (2014a) Mental health Atlas country profile 2014. Available at: http://www.who.int/mental_health/evidence/atlas/mental_health_atlas_2014/en/.

[10] Akbari H Majdzadeh R Foroushani AR Raeisi A (2013) Timeliness of malaria surveillance system in Iran. Iran J Public Health. 42: 39– 47. 23515191 PMC3595634

[11] Hatam GR Nejati F Mohammadzadeh T Shahriari-Rad R Sarkari B (2015) Population based seroprevalence of malaria in Hormozgan Province, Southeastern Iran: A low transmission area. Malar Res Treat. 2015: 174570. 26543662 10.1155/2015/174570 PMC4620240

[12] Soleimani-Ahmadi M Vatandoost H Zare M Turki H Alizadeh A (2015) Topo-graphical distribution of Anopheline mosquitoes in an area under elimination programme in the south of Iran. Malar J. 14: 262. 26148647 10.1186/s12936-015-0771-7 PMC4491864

[13] Nejati J Vatandoost H Oshaghi MA Salehi M Mozafari E Moosa-Kazemi SH (2013) Some ecological attributes of malarial vector *Anopheles superpictus* Grassi in endemic foci in Southeastern Iran. Asian Pac J Trop Biomed. 3( 12): 1003– 1008. 24093794 10.1016/S2221-1691(13)60193-8 PMC3805098

[14] World Health Organization (2015b) World Malaria Report 2015. WHO Press, Switzerland.

[15] Azari-Hamidian S (2007) Checklist of Iranian mosquitoes (Diptera: Culicidae). J Vector Ecol. 32( 2): 235– 242. 18260513 10.3376/1081-1710(2007)32[235:coimdc]2.0.co;2

[16] Mehravaran A Vatandoost H Oshaghi MA Abai MR Edalat H Javadian E Mashayekhi M Piazak N Hanafi-Bojd AA (2012) Ecology of *Anopheles stephensi* in a malarious area, southeast of Iran. Acta Med Iran. 50( 1): 61– 65. 22267381

[17] Alegana VA Wright JA Nahzat SM Butt W Sediqi AW Habib N Snow Robert W. Atkinson Peter M. Noor Abdisalan M. (2014) Modeling the incidence of *Plasmodium vivax* and *Plasmodium falciparum* malaria in Afghanistan 2006–2009. PLoS One. 9( 7): e102304. 25033452 10.1371/journal.pone.0102304 PMC4102516

[18] Leslie T Mohammed N Omar H Rasheed HU Vorst F Sediqi AM (2008) Malaria sentinel surveillance in Afghanistan. Afghan Annu Malar J. 1: 114– 128.

[19] Peragallo MS Sarnicola G Boccolini D Romi R Mammana G (2014) Risk assessment and prevention of malaria among Italian troops in Afghanistan, 2002 to 2011. J Travel Med. 21( 1): 24– 32. 24383651 10.1111/jtm.12046

[20] Adimi F Soebiyanto RP Safi N Kiang R (2010) Towards malaria risk prediction in Afghanistan using remote sensing. Malar J. 9: 125. 20465824 10.1186/1475-2875-9-125 PMC2878304

[21] Kassiri H Javadian E Hanafi-Bojd AA (2011) Faunistic survey of sandflies (Diptera: Psychodidae) in Chabahar county, Southeast of Iran. J Exp Zool India. 14( 2): 663– 666.

[22] Shahgudian ER (1960) A key to the anophelines of Iran. Acta Med Iran. 3: 38– 48. 13911134

[23] World Health Organization (2015a) Overview of malaria elimination. Available at: http://www.who.int/malaria/areas/elimination/overview/en/.

[24] Oyewole IO Ibidapo CA Okwa OO Oduola AO Adeoye GO Okoh HI Awolola TS (2010) Species composition and role of *Anopheles* mosquitoes in Malaria transmission along Badagry axis of Lagos Lagoon, Lagos, Nigeria. Int J Insect Sci. 2: 51– 57.

[25] Oshaghi MA Vatandoost A Gorouhi A Abai MR Madjidpour A Arshi S Sadeghi H Nazari M Mehravaran A (2011) Anopheline species composition in borderline of Iran-Azerbaijan. Acta Trop. 119: 44– 49. 21513694 10.1016/j.actatropica.2011.04.005

[26] Manouchehri AV Zaim M Emadi AM (1992) A review of malaria in Iran, 1957–1990. J Am Mosq Control Assoc. 8( 4): 381– 385. 1474383

[27] Mehdipour D Moosa-Kazemi SH Hosseini M Zolfi R (2013) Some ecological aspects of malaria vectors in saravan area, Iran. Arch Hyg Sci. 2( 1): 31– 40.

[28] Edalat H Moosa-Kazemi SH Abolghasemi E Khairandish S (2015) Vectorial capacity and age determination of *Anopheles stephens* Liston (Diptera: Culicidae), during the malaria transmission in Southern Iran. J Entomol Zool Stud. 3( 1): 256– 263.

[29] Soleimani-Ahmadi M Vatandoost H Shaeghi M Raeisi A Abedi F Eshraghian MR Madani A Safari R Shahi M Mojahedi A Poorahmad-Garbandi F (2012) Vector ecology and susceptibility in a malaria endemic focus in southern Islamic Republic of Iran. East Mediterr Health J. 18: 1034– 1041. 23301358 10.26719/2012.18.10.1034

[30] Salahi-Moghadam A Khoshdel AR Barati M Sedagbat MM (2014) An overview and mapping of Malaria and its vectors in Iran. Hormoz Med J. 18( 5): 473– 485.

[31] Barik TK Sahu B Swain V (2009) A review on *Anopheles culicifacies*: from bionomics to control with special reference to Indian subcontinent. Acta Trop. 109: 87– 97. 19000647 10.1016/j.actatropica.2008.09.017

[32] Kadamov DS (2006) The bloodsucking mosquitoes (Culicidae) of North Afghanistan. Med Parazitol (Mosk). (1): 59– 60. 16562755

[33] Brooker S Leslie T Kolaczinski K Mohsen E Mehboob N Saleheen S Khudonazarov J Freeman T Clements A Rowland M Kolaczinski J (2006) Spatial epidemiology of *Plasmodium vivax*, Afghanistan. Emerg Infect Dis. 12( 10): 1600– 1602. 17176583 10.3201/eid1210.060051 PMC1773016

[34] Ward RA (1972) Mosquito of Afghanistan –An annotated checklist. Mosq Syst. 4: 93– 97.

[35] Ahmad M Buhler C Pignatelli P Ranson H Nahzat SM Naseem M Farooq Sabawoon M Siddiqi AM Vink M (2016) Status of insecticide resistance in high risk malaria provinces in Afghanistan. Malar J. 15: 98. 26888409 10.1186/s12936-016-1149-1 PMC4758152

[36] Oshaghi MA Yaghobi-Ershadi MR Shemshad K Pedram M Amani H (2008) The *Anopheles superpictus* complex: introduction of a new malaria vector complex in Iran. Bull Soc Pathol Exot. 101: 429– 434. 19192616

[37] Harbach RE (2004) The classification of genus *Anopheles* (Diptera: Culicidae): a working hypothesis of phylogenetic relationships. Bull Entomol Res. 94( 6): 537– 553. 15541193 10.1079/ber2004321

[38] Hwang UW (2007) Revisited ITS2 phylogeny of *Anopheles (Anopheles) Hyrcanus* group mosquitoes: reexamination of unidentified and misidentified ITS2 sequences. Parasitol Res. 101( 4): 885– 894. 17514381 10.1007/s00436-007-0553-4

[39] Ponçon N Toty C Kengne P Alten B Fontenille D (2008) Molecular evidence for similarity between *Anopheles hyrcanus* (Diptera: Culicidae) and *Anopheles pseudopictus* (Diptera: Culicidae), sympatric potential vectors of malaria in France. J Med Entomol. 45( 3): 576– 580. 18533455 10.1603/0022-2585(2008)45[576:mefsba]2.0.co;2

[40] Azari-Hamidian S Abai MR Ladonni H Vatandoost H Akbarzadeh K (2006) *Anopheles peditaeniatus* (Leicester) new to the Iranian mosquito fauna with notes on *Anopheles hyrcanus* group in Iran. J Am Mosq Control Assoc. 22( 1): 144– 146. 16646339 10.2987/8756-971X(2006)22[144:APLNTT]2.0.CO;2

[41] Djadid ND Jazayeri H Gholizadeh S Rad ShP Zakeri S (2009) First record of a new member of *Anopheles hyrcanus* Group from Iran: molecular identification, diagnosis, phylogeny, status of kdr resistance and *Plasmodium* infection. J Med Entomol. 46( 5): 1084– 1093. 19769039 10.1603/033.046.0515

